# Transient mutation bias increases the predictability of evolution on an empirical genotype–phenotype landscape

**DOI:** 10.1098/rstb.2022.0043

**Published:** 2023-05-22

**Authors:** James S. Horton, Shani U. P. Ali, Tiffany B. Taylor

**Affiliations:** Milner Centre for Evolution, Department of Life Sciences, University of Bath, Claverton Down, Bath BA2 7AY, UK

**Keywords:** mutation bias, mutational hotspot, genotype–phenotype landscape, experimental evolution, historical contingency, epistasis

## Abstract

Predicting how a population will likely navigate a genotype–phenotype landscape requires consideration of selection in combination with mutation bias, which can skew the likelihood of following a particular trajectory. Strong and persistent directional selection can drive populations to ascend toward a peak. However, with a greater number of peaks and more routes to reach them, adaptation inevitably becomes less predictable. Transient mutation bias, which operates only on one mutational step, can influence landscape navigability by biasing the mutational trajectory early in the adaptive walk. This sets an evolving population upon a particular path, constraining the number of accessible routes and making certain peaks and routes more likely to be realized than others. In this work, we employ a model system to investigate whether such transient mutation bias can reliably and predictably place populations on a mutational trajectory to the strongest selective phenotype or usher populations to realize inferior phenotypic outcomes. For this we use motile mutants evolved from ancestrally non-motile variants of the microbe *Pseudomonas fluorescens* SBW25, of which one trajectory exhibits significant mutation bias. Using this system, we elucidate an empirical genotype–phenotype landscape, where the hill-climbing process represents increasing strength of the motility phenotype, to reveal that transient mutation bias can facilitate rapid and predictable ascension to the strongest observed phenotype in place of equivalent and inferior trajectories.

This article is part of the theme issue ‘Interdisciplinary approaches to predicting evolutionary biology’.

## 1. Introduction

Adaptive landscapes have the potential to become powerful tools for predicting evolution [1]. Persistent selection under a constant environment confines the viable mutational space available to populations, restricting their ability to acquire neutral or deleterious changes that would move them ‘sideways’ or ‘downwards’ on the landscape and instead drives them upward to realize the local fitness optimum [2]. This means that once an adapting population has set upon a path, possessing complete knowledge of the landscape and adaptive context can allow an observer to predict which trajectories are more likely to be taken [3]. And by extension, it can also help them to determine which peak(s) a population will most likely ascend toward, allowing them to forecast genotypes and resultant phenotypes [1] with improved success.

Navigating across a landscape offers two key features of interest—the peak that a population eventually reaches, and the route it takes to get there [4]. While selection tends to drive populations toward adaptive peaks, the mutational trajectory taken (i.e. the route travelled) is much less predictable [5]. However, mutational biases can enhance predictability for both the trajectory and the realized peak. Genome-wide mutational biases—such as transition or transversion biases—have been shown to impact navigability across landscapes [6,7]. Mutation biases can influence mutational trajectories to drive populations toward sub-optimal peaks [8], and the reversal of mutation biases can unlock pathways to higher peaks [9]. Whether bias ushers populations toward optimality can be determined by the environment, as environmental changes can alter landscape topography and shift whether the biased route leads to an optimal or sub-optimal peak [10]. Localized mutation biases—which act to raise the mutation rate of one mutation within an adaptive spectrum, generating a ‘mutational hotspot’ [11]—act to influence which mutational trajectory an adapting population will ascend by setting it upon a particular path. By understanding the trajectory, we can anticipate the mutational pathway navigated by a population and acquire genetic resolution to aide our evolutionary predictions.

Adaptive evolution is ‘short-sighted’, but single-step mutations can elicit long-term consequences [12]. This concept is termed historical contingency [13], which describes that the future of a genome's evolution is dependent on its current genomic background [14]. Genome-wide mutational biases have a persistent impact on trajectories throughout all mutational events during the ascent of a landscape [15], although the emergence of mutators during adaptation can remove or reverse these biases [16]. However, localized hotspots, which act on a singular adaptive position, can be lost after a single round of mutation. Yet if a hotspot acts early in the adaptive walk, this transient mutation bias can sway ascension toward a particular adaptive trajectory. And in doing so, it can possibly confine subsequent mutational steps to remain on that path [12,17].

As evolution is short-sighted, such biases that operate transiently are likely not adaptive with regard to the peak that is eventually realized following successive mutations. But while they may not have evolved under positive selection, hotspots may still have adaptive value. In some circumstances, a neutrally evolved mutational hotspot may facilitate rapid advancement toward a sub-optimal peak, or it may instead usher a population toward a competitive fitness peak via a predictable trajectory. This latter potential is particularly interesting with regard to predicting evolution, as in this scenario localized mutation bias can help to enhance and refine predictions based on selection alone. Although the role of mutation bias in navigating adaptive landscapes has been explored theoretically, empirical data in this area are lacking. In this work, we set out to experimentally examine the role of potent transient mutation bias in realized adaptive outcomes.

We use engineered immotile variants of the soil bacterium *Pseudomonas fluorescens* SBW25 (labelled AR2), which are without functionality of *viscB* (required for biosurfactant-mediated motility) and the master regulator of flagellar motility, *fleQ* [18]. Populations harbouring this genotype can, however, rapidly recover a strong flagellar-mediated motility phenotype through a two-step (i.e. two mutation) evolutionary process [19]. This process predominantly targets genes within the nitrogen regulatory (ntr) pathway containing four mutable loci; the first mutational step (at any of the four loci) recovers motility, and the second mutational step (occurring within the gene *ntrC*) refines the phenotype and bolsters motility yet further to near wild-type levels [19]. This empirical model therefore presents a small, structured genotype–phenotype space whereby the strength of the motility phenotype can be mapped over a two-step adaptive walk involving mutational targets at multiple positions within a collection of four loci.

This system additionally possesses a potent mutational hotspot that heavily biases populations to realize identical first-step mutations [11]. One of the possible mutations granting motility is a single transversion mutation A→C at position 289 (A289C) within the *ntrB* gene, which encodes the histidine kinase that controls NtrC activity. For AR2 populations under selection for motility *ntrB* A289C is repeatedly realized, appearing in approximately 95% of independently evolving populations despite other mutations offering comparable motility phenotypes [11].

Due to this potent localized mutation bias, uncovering other viable targets that comprise the spectrum of the first-step mutations is challenging. However, in previous work, we have demonstrated that the hotspot can be removed through functionally benign genomic augmentations, such as silent mutations [11] and gene strandedness [20]. This has allowed us to capture rare mutational targets that did not have the opportunity to be realized when the 289 hotspot remains intact. Now armed with a more complete spectrum of mutational targets that represent possible first adaptive steps, we were able to investigate the adaptive consequences of a localized mutation bias that severely constrains the spectrum of realized first-step mutations. We sought to understand whether the mutational hotspot would usher genotypes to realize an inferior phenotype, or if it could reinforce the predictability of landscape navigability offered by selection by reliably driving populations to realize the strongest motility phenotype via a predictable mutational trajectory. We addressed this question by isolating and evolving the spectrum of first-step mutants, allowing them to complete the short walk toward the peak of their adaptive phenotypes and analysing whether those with a biased first step ascended to the highest phenotypic outcome.

## 2. Methods

### (a) Bacterial strains

All strains used in this study were derived from an immotile variant of *Pseudomonas fluorescens* SBW25 that has a partial gene deletion in *fleQ*—the master regulator for flagellar motility—and a transposon-insertion in the gene *viscB*, which facilitates an alternative surface-spreading motility phenotype. This strain (SBW25*ΔfleQ* IS-ΩKm-hah: *PFLU2552*), dubbed ‘AR2’, is therefore rendered completely immotile [18]. Two additional genotypes were engineered from the AR2 genomic background prior to evolution experiments. The first genotype, AR2-sm, harbours six synonymous changes within the locus *ntrB*: C276G, C279T, C285G, C291G, T294G and G300C [11]. The second genotype, AR2 *ΔntrBC* Tn7-*ntrBC*-sm-lead (hereafter AR2 Tn7), has undergone a deletion of the native *ntrBC* operon that is replaced by a Tn7 mini-transposon-mediated integration of the *ntrBC* operon (and its accompanying promoter and terminator regions) downstream of the locus *glmS* [20]. This integrated *ntrBC* operon possesses the six synonymous changes and is encoded onto the leading replicative strand. Relative to their AR2 ancestor, these genomic augmentations are functionally benign but do affect the potency of the mutational hotspot operating at nucleotide position 289 within the *ntrB* locus [11,20]. The changes therefore allowed for the appearance and isolation of rare novel adaptive motile genotypes in previous work that were previously unseen in AR2 strains. The genomic backgrounds for the first-step motile genotypes used in this study are as follows, AR2: *ntrB* A289C, *ntrB* A683C, *ntrB Δ*406-417; AR2-sm: *ntrB Δ*93-104, *glnK* A5C; AR2 Tn7: *glnK* 5886 del, *glnA* T169A, *ntrC* C251A.

### (b) Evolution experiments

Populations were placed under selection for the flagellar motility phenotype by allowing them to grow for 7 days (approx. 168 h) on extra wide circular Petri dishes (Nunc™, 140 mm diameter) containing soft agar (0.25%) supplemented with lysogeny broth (LB). These motility plates were prepared by pouring 76 ml of molten soft agar into each Petri dish, replacing the lid, and leaving the plates to dry for at least 4 h to no longer than 24 h before removing the lids and drying for 30 min prior to inoculation. Multiple biological replicates were prepared for each starting genotype, with a single biological replicate used to inoculate no more than four dishes. Overnight cultures of replicates were corrected to a cell density of OD_595_ = 1 unit / ml, and 1 µl of the corrected mix was then inoculated into the centre of the dish. This was achieved by using the pipette tip to puncture approximately 1 mm into the surface of the agar from a perpendicular position and ejecting the culture into the cavity as the pipette was withdrawn. The motile first-step genotypes initially grow outward in a concentric circle, but emergent second-step mutants ‘bleb’ from the outer edges and often generate uneven, undulating frontiers. At the end of the experiment, a sample was harvested from the leading edge of the frontier that had migrated furthest from the inoculation site. This was done by touching an inoculating loop against the frontier and passaging onto an LB agar plate so that a single colony could be isolated. This single colony was used to seed a single overnight culture that would subsequently provide template DNA for sequencing, the population used for phenotyping motility and the population stored cryogenically.

In instances where two distinct frontiers had travelled equal distances (within approximately 2 mm) from the central inoculation site, two samples were isolated per dish. If populations reached the edge of the dish prior to 168 h, a sample was taken from the agar's edge at this earlier timepoint. Evolving populations were discarded from the study during the evolution experiment if motile satellite populations of *P. fluorescens* were observed, or if contamination from other species was observed to have encountered the outwardly migrating motile zone. Samples were also discarded if adapted overnight cultures did not grow to a reasonable optical density (approx. OD_595_ = 0.5) within 48 h, as these populations were highly susceptible to fixing compensatory mutations that removed motility, leaving us unable to quantify the motility phenotype.

### (c) Measuring motility

The motility phenotype was measured by allowing motile populations to migrate across standard 88 mm diameter Petri dishes containing 0.25% soft agar supplemented with LB for 24 h. Inoculating population sizes were standardized, and dishes were inoculated as described above. Dishes were also prepared as stated above, with the exception that 30 ml were used to fill the standard Petri dishes. As motility zones had been observed to vary between batches of soft agar, the motile zones of all populations were standardized against a mutant harbouring the hotspot mutation *ntrB* A289C that was assessed within the same batch. All mutants had at least three technical repeats. For those where a larger range in data was observed, the assay was repeated with an additional biological replicate of six technical repeats.

### (d) Sequencing

Second-step mutations targeting the ntr pathway were identified through PCR amplification and sequencing of the *ntrC* helix-turn-helix domain using primer sequences: 5′-GGATGGCGAGTTCTATCGGG-3 and 5′-CGGTTCATGGTGCATTGAAGC-3′. Samples were prepared using a Monarch® PCR Cleanup kit (NEB) and Sanger sequencing was performed by Eurofins Genomics. If two genotypes were taken from the same motile plate and were observed to have the same mutation in *ntrC*, these were deemed to have arisen from the same sub-population and treated as one isolate. Following initial phenotyping, a smaller subset of mutants showcasing weak, median, and strong motility from genomic backgrounds *ntrB* A289C, *glnK* A5C, *glnK* 5886 del., *ntrC* C251A and *glnA* T169A (no median mutant sent for *glnA*) were sent for Illumina whole-genome sequencing (WGS). Genome resequencing was performed by SeqCenter, and single-nucleotide variants and small indels were called using Snippy with default parameters [21], using *P. fluorescens* SBW25 genome as an assembly template (NCBI Assembly: ASM922v1, GenBank sequence: AM181176.4). This analysis was performed through the Cloud Infrastructure for Microbial Bioinformatics (CLIMB) [22].

### (e) Data visualization and statistics

All statistical tests were performed in R. Comparisons between two groups were performed using statistical tests in base-R (significant *p* ≤ 0.05): Shapiro–Wilks normality tests were performed followed by unpaired *t*-tests or Wilcoxon rank-sum tests with continuity correction for non-normally distributed data. Group counts were compared using a Pearson's chi-squared test. When replicate data points were added through repeated assays, Dixon tests were performed to determine any potential outliers, but none were found. Pairwise comparisons across more than two groups used the Dunn.test package (significant *p* ≤ 0.025): a Kruskal–Wallis test followed by *post hoc* Dunn test and Benjamini–Hochberg correction were performed. Data in figure 4 were visualized using plotFitnessLandscape from the OncoSimulR R package, which is based on the landscape visualization tool MAGELLAN [23].

## 3. Results

**Figure 1.**
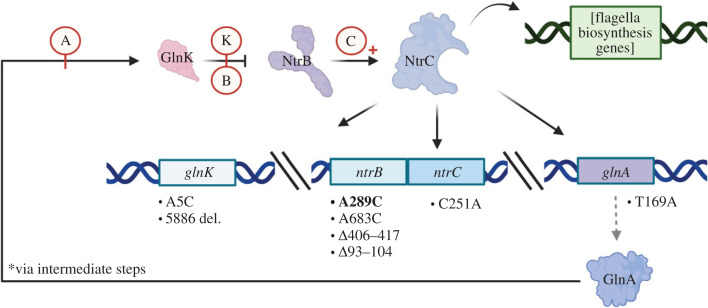
A simplified nitrogen regulatory (ntr) pathway schematic focusing on four key loci and their protein products, demonstrating how a strong motility phenotype can be achieved following a two-step adaptive walk in AR2 lines. Black arrows denote either positive activation of a protein or transcriptional activation of a locus/operon by the transcription factor NtrC. The blunted arrow pointing from GlnK to NtrB denotes regulatory suppression. First-step mutations that restore motility are shown beneath the genes where the mutations are found. These mutations are believed to functionally perturb or break network interactions leading to the over-activation of NtrC: Mutation within *glnA* disrupts glutamine synthesis leading to the downstream inactivation of GlnK (anno. A). Mutations impacting *glnK* and *ntrB* prevent GlnK suppression of NtrB phosphorylation activity (annos. K and B). Mutation within *ntrC*'s receiver domain may perturb NtrB–GlnK de-phosphorylation activity (anno. C). The second-step mutation is often observed within *ntrC*'s helix-turn-helix domain [19], which bolsters binding affinity for genes of the flagellar network (curved arrow). The hotspot mutation is highlighted in bold. Regulatory connections derived from publications by [19,24–26]. Figure created with BioRender.com.

The small empirical genotype–phenotype landscape investigated in this work centres on loci of the nitrogen regulatory pathway, which can mutate to recover and bolster flagellar-mediated motility in aflagellate strains of *P. fluorescens* SBW25 (figure 1). This two-step adaptive walk centres on four loci of interest: (i) *glnK*, which encodes a negative regulator of the histidine kinase NtrB's phosphorylation activity; (ii) *ntrB*, which encodes the histidine kinase that regulates activity of the response regulator, NtrC; and (iii) *ntrC*, which encodes the response regulator of the nitrogen pathway. NtrC is a homologue of the flagellar master regulator FleQ, and therefore when hyper-phosphorylated can act as a surrogate for FleQ-dependent expression [19]. This hyper-phosphorylation can be achieved through mutation in either *glnK*, *ntrB*, *ntrC*, or through mutation in (iv) *glnA*, which encodes glutamine synthetase—a major nitrogen regulatory protein regulated by NtrC. *glnA* offers an indirect route to NtrC hyper-phosphorylation by altering the physiological nitrogen balance within the cell [19], leading to reduced GlnK-mediated repression and increased NtrBC activity (figure 1). *glnK*, *ntrB* and *ntrC* instead directly increase activity through loss-of-function mutations that likely de-sensitize the response regulator from negative regulation of the network (figure 1). The second step of the adaptive walk in the ntr pathway involves mutation within the helix-turn-helix domain-encoding region of *ntrC*. This mutation switches affinity from NtrC's native regulatory binding sites to the flagellar pathway [19]. This mutation is in all likelihood only adaptive after the first step (previously described as a ‘refining mutation’, [27]) and neutral prior (sign epistasis), which would explain why we are yet to see such a mutation during a first adaptive step [11,19,20].

While mutations within any of the four loci can grant the motility phenotype [11,20], only a small number of these mutations are usually recovered due to a mutational hotspot at nucleotide position 289 within the locus *ntrB*. However, through functionally benign genomic augmentation, we were able to collect a suite of first-step mutants (this work; [11,20]), out of which 8 were chosen for further investigation of their adaptive trajectories (figure 1). These include the hotspot mutant *ntrB* A289C, and alternative mutations within *ntrB* including the second-most frequently observed mutation *Δ*407-416, an alternative A→C transversion A683C and a rare deletion *Δ*93–104. We also investigated lines with single-nucleotide variations in *glnK* (A5C), *glnA* (T169A) and *ntrC*'s receiver domain (C251A). Finally, we included a rare large deletion event of 5886 nucleotides that entirely removed *glnK*, another gene of the nitrogen pathway *amtB* (*PFLU5952*) and several neighbouring genes. We sought to understand if the mutational trajectory that was most likely to be followed due to mutation bias at the first step led to superior or inferior motility phenotypes following subsequent adaptive mutations, relative to the mutational trajectories that are less likely to be taken.

### (a) The second step of the adaptive walk within the nitrogen regulatory pathway is more accessible to first-step *ntrB* and *glnK* mutants

**Figure 2.**
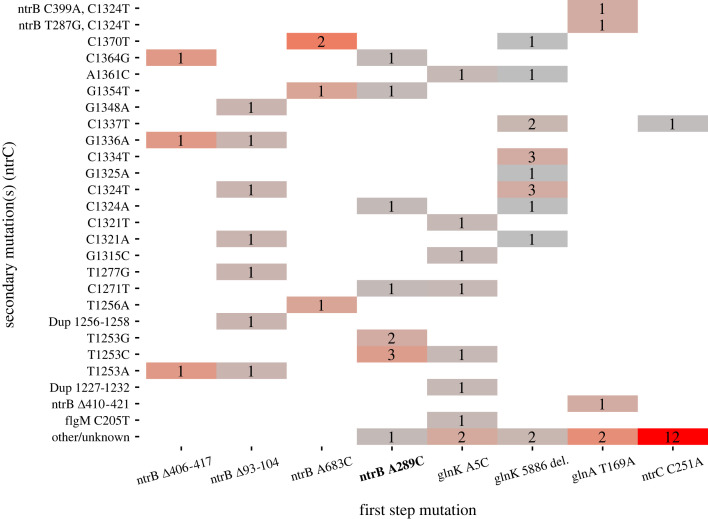
Frequency of identified mutations following selection for improved motility shows that *ntrB* and *glnK* mutants more readily adapt via a second-step mutation in *ntrC*. Genetic backgrounds containing first-step mutations are shown on the *x*-axis, with the hotspot mutation highlighted in bold. The frequency of each mutation observed within their descendants is shown in single cells corresponding to their description on the *y*-axis; triple mutants show two secondary mutations separated by a comma. Frequencies are coloured from a scale of grey (low frequency) to red (high frequency). ‘Other/unknown’ lines reported WT *ntrC* sequences following Sanger sequencing (*n* = 15) or identified no second-step mutations following WGS (*n* = 4). Unless otherwise stated, single-nucleotide variants and indels were observed at the described nucleotide positions within the locus *ntrC*. Number of replicates (*n*) for each condition: *ntrB* A289C *n* = 10. Alternate *ntrB* mutations *Δ*407–416 *n* = 3, *ntrB Δ*93–104 *n* = 7, *ntrB* A683C *n* = 4. *glnK* A5C *n* = 9, *glnK* 5886 del. *n* = 15. *glnA* T169A *n* = 5. *ntrC* C251A *n* = 13.

Our experimental regime involved allowing multiple replicate lineages for each first-step genotype to grow for 7 days (approx. 168 h) under selection for motility using extra wide Petri dishes holding soft LB agar (0.25%). At the end of this experiment, an isolated genotype from the sub-population that had migrated furthest on the plate was isolated and a large subset of these were sequenced. Most lineages used amplicon sequencing at the locus *ntrC*'s C-terminus containing the helix-turn-helix domain, but a smaller subset (*n* = 14, see §2) was sent for WGS, which allowed us to identify adaptive mutations outside of this locus. The frequencies of the second-step mutations for each starting lineage are shown in figure 2.

Of the 66 sequenced mutants, we observed a spectrum of 22 unique *ntrC* mutations, and mutations within the helix-turn-helix encoding region were the most frequently observed targets for all *ntrB* and *glnK* first-step lineages (figure 2). One second-step mutation from a *glnK* A5C lineage was independent of ntr-based adaptation, instead being observed within the gene *flgM* (C205T), which is a negative regulator in the flagellin cascade. Multiple populations descended from *glnA* T169A also harboured mutations outside of *ntrC*, but these occurred within the ntr pathway within the locus *ntrB*. One lineage harboured only *ntrB Δ*410–421, which produces the same protein product as *ntrB Δ*406–417 and has been observed in previous work [11]. The other two lineages harboured previously unseen *ntrB* single-nucleotide variants alongside the *ntrC* mutation C1324T (figure 2). Therefore, *ntrC* mutation was only observed when *ntrB* mutation was also present in *glnA* descended lines. This suggests that *glnA* mutants require a three-step evolutionary pathway, via *ntrB*, to reach a similar phenotypic strength. Other than these identified mutations, 2/5 of *glnA* T169A harboured no identified mutations in *ntrC*. This lack of *ntrC* evolvability was more pronounced in *ntrC* C251A populations, where only one evolved population possessed a *ntrC* helix-turn-helix mutation. The remaining approximately 92% (12/13) harboured no additional identified mutations (figure 2), displaying significantly lower ntr-based evolution than the hotspot mutant *ntrB* A289C (chi-square, *p* < 0.0001). These lines therefore harboured alternative second-step mutations outside the locus or simply did not evolve in the allotted timeframe of the experiment.

There are two caveats to these results. First, all evolved populations were granted the same amount of time to evolve, and as such this does not control for differences in generation time. No severe differences in growth rate were observed during routine culturing for *ntrB*, *glnK* and *ntrC* C251A mutants, and as such it is likely that elapsed generations (and the opportunity for mutation) were roughly comparable across these populations. However, *glnA* T169A was observed to exhibit severe pleiotropy during routine culturing, with a prominent fitness penalty when growing in liquid media. Populations evolving with this mutation therefore likely went through fewer generations throughout the course of the experiment and therefore had a smaller evolving population size. Despite this, *ntrC* C251A still had the fewest positively identified *ntrC* second-step mutations. Second, *glnK* 5886 del., *ntrC* C251A and *glnA* T169A are from a Tn7 background wherein the *ntrBC* operon has been translocated to a new genomic position and re-orientated onto the leading strand. This therefore may have had a bearing on the mutability of the *ntrC* locus in this new genomic position, relative to *ntrB* A289C mutants that possess the *ntrBC* operon in its native position. However, *glnK* 5886 del. populations proved highly evolvable throughout the course of the experiment, which provides reassurance that the translocation of *ntrC* does not explain the lack of evolvability observed in *ntrC* C251A-derived lines. Therefore, other factors are required to explain the lack of further adaptation for *ntrC* C251A.

### (b) Motile lineages descended from hotspot mutation *ntrB* A289C offer equivalent or superior motility phenotypes compared to alternative mutational routes

**Figure 3.**
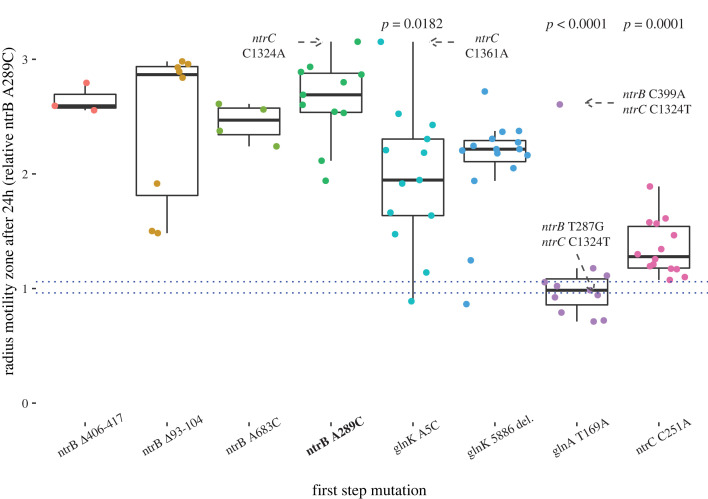
Motile lineages descended from hotspot mutation *ntrB* A289C offer equivalent or superior motility phenotypes compared to alternative mutational routes. Motile first-step ancestors are shown on the *x*-axis, with the hotspot mutation highlighted in bold. The radius of the motility zones, relative to hotspot mutation *ntrB* A289C first-step mutation, for the spectrum of descendants is shown on the *y*-axis. Each point represents the median phenotypic value of at least three technical replicates for each isolated descendent (mean range = 0.17; technical repeat data available in electronic supplementary material, Data, table S1). Boxes display the median, and first to third interquartile range (IQR) for the median phenotypic values. Whiskers show outside values up to 1.5 x IQR. First and third IQR for the *ntrB* A289C first-step control replicates are denoted by dotted blue lines. The *p*-values (Dunn test) for descendent populations with significantly different distributions of motility relative to *ntrB* A289C descendants are displayed above the relevant lineages. The strongest second-step genotypes, and the identified triple mutants, are annotated on the figure. Number of replicates (*n*) for each condition: *ntrB* A289C *n* = 11. Alternate *ntrB* mutations *Δ*406–417 *n* = 3, A683C *n* = 4, *Δ*93–104 *n* = 8. *glnK* A5C *n* = 13, *glnK* 5886 del. *n* = 15. *glnA* T169A *n* = 11. *ntrC* C251A *n* = 14.

After isolating a library of first-step descendants, we next quantified their motility to assess which genotypes encoded the strongest phenotype. As described in figure 2, descendants of *ntrB* and *glnK* mutants reliably acquired mutations within *ntrC* throughout the course of the experiment. These mutations were found to offer the strongest motility phenotypes in the collected dataset (figure 3). We additionally did not observe a strong epistatic signal between the spectrum of *ntrC* mutations observed and the initial first-step mutation, with all two-step ntr mutant spectra proving non-significantly different from the *ntrB* A289C, *ntrC* mutant spectra (Dunn test, *p* range = 0.0490–0.4704). The exception to this was in *glnA* T169A descendants, where both *ntrC* mutants also harboured an additional mutation in *ntrB*, thus requiring three mutational steps overall.

Descendants of the *ntrB* A289C-biased trajectory therefore did not achieve stronger motility phenotypes than alternative two-step ntr mutants, but neither was their motility inferior to any observed alternative route. This remained true when focusing on the strongest motility genotypes—*ntrB* A289C, *ntrC* C1324A and *glnK* A5C, *ntrC* A1361C—which could not be distinguished phenotypically (T-test, *p* = 0.5489). However, when the analysed dataset is expanded to include non-ntr evolution (inclusive of alternative adaptive pathways, undetermined mutations and potentially unevolved genotypes, shown in figure 3), the proficiency of hotspot *ntrB* A289C being able to reliably and rapidly acquire *ntrC* mutation produces a more competitive spectrum. Overall, *ntrB* A289C evolved a phenotypically stronger collection of descendants than *glnA* T169A (Dunn test, *p* *<* 0.0001), *ntrC* C251 (Dunn test, *p* *=* 0.0002) and *glnK* A5C (Dunn test, *p* = 0.0182); and a non-significantly different collection of phenotypes to *glnK* 5886 del. (Dunn test, *p* = 0.0374), and alternative *ntrB* mutants (Dunn test, *p* range = 0.3915–0.5111).

The reason for the superior spectrum of *ntrB* A289C descendants is either due to alternative first-step mutations ascending alternate, inferior trajectories, or due to impaired navigability in the other first-step loci. These differences were most prominent between the hotspot mutation and first-step mutations in *ntrC* and *glnA.* Although the genetic and molecular reasons are unclear, *ntrC* C251A populations repeatedly failed to yield descendants that could compete with the strongest phenotypes. As such we can infer that navigability upward through the landscape from this genotype is restricted, perhaps due to a limited number of viable mutational targets in the helix-turn-helix domain when a mutation has already occurred at position 251. However, *ntrC* C251A mutants can attain comparable motility via *ntrC* helix-turn-helix mutation, as the mutant *ntrC* C251A, C1337T is not significantly outperformed by *glnK* first-step mutants with the same *ntrC* mutation (*glnK* 5886 del., *ntrC* C1337; T-test, *p* = 0.1467).

**Figure 4.**
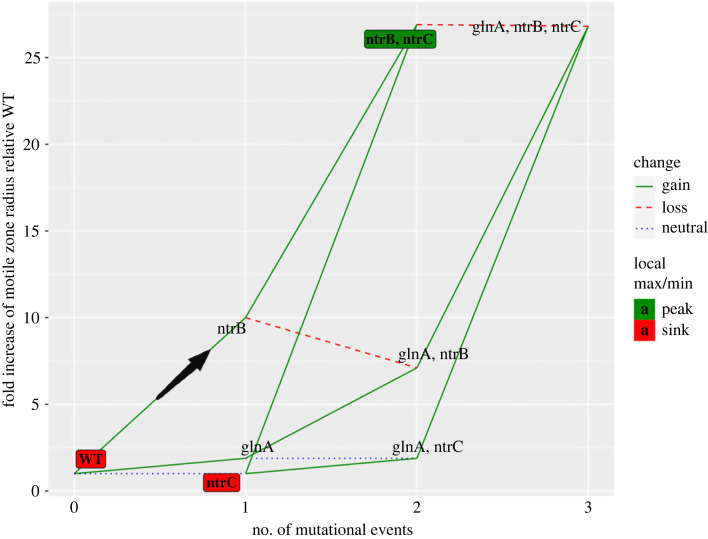
Following a biased trajectory allows adapting populations to reach the strongest observed phenotype in the fewest mutational steps. A genotype–phenotype landscape detailing phenotypic trajectories based on mutation within three loci: the hotspot-harbouring locus *ntrB*, *glnA* and *ntrC* (helix-turn-helix encoding domain). The point where two lines meet denotes a mutant genotype (the annotation) and its associated phenotypic strength (*y*-axis position) after a given number of mutational events from the ancestral AR2 genotype (*x*-axis position). Edges connecting the annotations represent genotypes connected by one mutational event. The biased mutational step is labelled with a black arrow. Unless otherwise stated, motile zone radii (*y*-axis) are derived from mean radius values for the following mutations: WT (ancestral genotype AR2) = no mutation, zone set to *ntrB* A289C radius/10 (immotile cells following 24 h growth on soft agar form colony with a small radius). *ntrB* = A289C. *glnA* = T169A. *ntrC* = helix-turn-helix first-step mutation not observed in this study or previous works [11,19,20] so inferred neutral, set equivalent phenotype to WT. *ntrB*, *ntrC* = A289C, median value of all A289C descended *ntrC* phenotypes. *glnA*, *ntrB* = T169A, *Δ*410-421. *glnA*, *ntrC* = *ntrC* inferred neutral, set equivalent phenotype to *glnA* T169A. *glnA*, *ntrB*, *ntrC* = T169A, C399A, C1324T (strongest observed *glnA* descendent phenotype shown).

*glnA* T169A descendants can reach a comparable phenotype to *ntrB* A289C descendants, permitted *ntrC* mutation is preceded by a mutation in *ntrB* (figure 3). An epistatic interaction may be present between the kinase and regulator in these lines, as the eventual phenotype achieved following *ntrC* C1324T mutation appears to be contingent on the specific *ntrB* mutation (figure 3). However, as only one of the triple mutants was sent for WGS, the epistatic interaction may instead be explained by an unidentified secondary mutation elsewhere in the genome. In either case, *glnA* descendants offer an inferior trajectory toward a strong motility phenotype relative to the hotspot trajectory. Depending on the *ntrB* mutation, evolving genotypes that acquire a *glnA* mutation prior to a *ntrB* mutation may confine the population to an inferior phenotype (figure 3) or require the evolving genotype to undergo three mutational steps to a comparable phenotype that is reliably achievable via two mutational steps when the hotspot mutation appears first (figure 4). A combination of *glnA* and *ntrB* mutation is phenotypically inferior to a singular *ntrB* mutation (Wilcox test, *p* = 0.0087), so a *glnA* mutation exhibits sign epistasis whereby it is beneficial from the ancestral immotile genotype but deleterious following mutation in *ntrB* (figure 4). Therefore, populations ascending via the biased trajectory are constrained to ascend to the strongest observed phenotype by both selection preventing deviations from the route and the number of remaining mutational steps needed to reach the highest point on the trajectory (figure 4).

Overall, these findings show that (i) ascension from the immotile ancestor to the strongest observed phenotype can be achieved by only two mutational events via the hotspot mutational route, (ii) 9/10 *ntrB* A289C populations evolved *ntrC* second-step mutations within 168 h, making it reliably evolvable and (iii) hotspot descendants showed the joint-strongest singular phenotype and evolved spectrum. Together these, respectively, show that the hotspot mutation *ntrB* A289C facilitates both rapid and reliable evolution to the strongest observed phenotype in place of inferior and comparable alternative mutational routes, meaning transient mutation bias can combine with selection to enhance the predictability of adaptive evolution.

## 4. Discussion

Adaptive landscapes have considerable potential as predictive tools in evolution [1]. Under strong selection that limits evolving populations from navigating ‘downward’ on adaptive slopes, sign epistasis between genotypes can generate multiple peaks that force populations to ascend toward the peak that they have already begun to climb [12,28,29]. Therefore selection can greatly restrict the number of viable mutational paths to high fitness from a much larger theoretical number [3]. However, selection is not the only actor at play as populations navigate landscapes. Mutation bias can be an ‘orientating factor’ in evolution [30]—it may ‘derail’ the expected outcome under selection and instead drive evolving populations to become stuck on sub-optimal peaks. Conversely, mutation bias may reinforce selection's optimal trajectory, drive populations away from inferior routes and shuttle them toward superior trajectories and higher peaks [8]. When multiple genetic routes can lead to an equivalent phenotype, mutation bias may make one trajectory more likely and therefore increase the resolution of our predictive power.

Such a finding was made in this study. We identified a number of first-step mutations that allowed previously immotile bacterial populations to recover flagella-mediated motility. These mutations each came from loci of the ntr pathway: either *glnK*, *glnA*, *ntrB* or *ntrC*, including one mutationally favoured trajectory—via *ntrB* A289C. We allowed multiple independent populations of each genotype to continue under selection for improved motility to determine which mutational trajectory led to the strongest motility phenotypes. We observed that mutational trajectories evolving from ancestors where first-step mutations were in *ntrC* and *glnA* offered inferior trajectories overall; these were less navigable, could confine populations to an inferior motility phenotype over the time course of the experiment, and required more mutational events to match the strongest observed phenotype. Conversely, mutations within the locus *ntrB*, including the hotspot mutation A289C, reliably offered the most mutationally accessible routes to the strongest observed phenotype. These routes could, however, be rivalled by mutations targeting the locus *glnK*, which mostly offered comparable phenotypes after the same number of mutations. This means that in a competitive adaptive setting free from mutation bias—in which selection will be the primary means to achieve predictable adaptive outcomes—a suite of first-step mutations across two loci would all be equally likely to reach fixation. However, with the bias from the hotspot working in conjunction with selection, the realized outcomes will be heavily constrained to one highly predictable mutational trajectory, providing increased genetic resolution for evolutionary predictions.

A challenge with empirical-based adaptive landscapes is that it is difficult to map phenotypes for the entire viable mutational spectrum, and therefore pertinent information may be missed in the unmapped terrain of the landscape. The ntr genotype–phenotype landscape is not entirely comprehensive, as the complete mutational spectrum for the evolution of flagellar motility can involve mutation outside of this pathway (e.g. *flgM* C205T, figure 2). However, first-step mutations outside the ntr pathway are only observed rarely after the A289C mutation bias is removed from *ntrB*, and more often but still rarely when the *ntrC* locus (and by extension ntr-based evolution) is removed entirely [31]. Furthermore, the first mutational step involving a non-ntr pathway takes far longer to evolve and offers significantly inferior motility relative to ntr mutants [31]. Also, the strongest phenotypes in this work belonged to *ntrC* second-step mutants (figure 3). As such, the genotype–phenotype landscape presented in this study captures common to rare mutational targets within the pathway that offers superior initial motility relative to alternative, rare pathways. This provides confidence that the phenotypes presented are the strongest that would be realized in a competitive adaptive setting.

The exploration of adaptive landscapes on evolutionary outcomes is largely a theoretical field, but complementary empirical systems that can reveal and explore ‘real’ adaptive landscapes are essential if we hope to implement and test theoretical outcomes [1,32]. An important revelation that microbial empirical studies have already revealed is that adaptive landscapes are rarely smooth, even in simple laboratory conditions [33]—but does that mean that the course of evolution is always unpredictable? By using a model system with a predictable evolutionary trajectory, such as one with access to a strong mutational hotspot, we can begin to explore features that can increase predictable outcomes, even in rugged landscapes, and understand the evolutionary consequences for constrained exploration of landscape space.

This work explores a small empirical genotype–phenotype landscape, within which a localized mutation hotspot acts on a single mutational step. Yet within this limited mutational space we can observe how a transient bias increases the predictive power of adaptive trajectories when placed in the context of a genotype–phenotype landscape. It may be that in other adaptive contexts, a similar ‘short-lived’ hotspot may drive populations toward an inferior trajectory or sub-optimal peak. Given that the population is large enough [34] and mutation is common enough [35], such a scenario may have a limited bearing on predicting evolution, as the hotspot genotypes will simply be outcompeted by less likely but more fit competitor mutations. In the ntr scenario, however, mutation bias complements selection, elevating the predictability of evolution to genetic resolution as one adaptive trajectory is repeatedly realized in place of comparable or inferior alternatives. This synergises with other research that described the likelihood of realizing a mutation is based on its impact on the protein product and the protein's position in the regulatory hierarchy [36], but that accurate forecasts using this knowledge can be impeded by missing information regarding hotspots [37]. Understanding hotspots may therefore prove especially useful for predicting navigability across other empirical landscapes that possess multiple mutational routes toward equally fit and equally mutationally accessible phenotypes [37]. Without bias it would be difficult to predict which trajectory would most likely be explored and therefore which mutations would reach fixation. But with a transient mutation hotspot that initiates the adaptive trajectory, accurate prediction can become markedly more achievable.

## Data Availability

The raw data for this study are available on the Open Science Framework (OSF), accessible at: https://osf.io/mg879/. The data are provided in the electronic supplementary material [38].
